# Observer variability for Lung-RADS categorisation of lung cancer screening CTs: impact on patient management

**DOI:** 10.1007/s00330-018-5599-4

**Published:** 2018-07-31

**Authors:** Sarah J. van Riel, Colin Jacobs, Ernst Th. Scholten, Rianne Wittenberg, Mathilde M. Winkler Wille, Bartjan de Hoop, Ralf Sprengers, Onno M. Mets, Bram Geurts, Mathias Prokop, Cornelia Schaefer-Prokop, Bram van Ginneken

**Affiliations:** 1 Department of Radiology and Nuclear Medicine, Radboud University Medical Center, Geert Grooteplein 10, 6525 GA Nijmegen, the Netherlands; 2 Department of Radiology, Vrije Universiteit Medical Center, Amsterdam, the Netherlands; 3 Department of Diagnostic Imaging, Section of Radiology, Nordsjællands Hospital, Hillerød, Denmark; 4 Department of Radiology, Streekziekenhuis Koningin Beatrix, Winterswijk, the Netherlands; 5 Department of Radiology, University Medical Center Utrecht, Utrecht, the Netherlands; 6 Department of Radiology, Academic Medical Center, Amsterdam, the Netherlands; 7 Department of Radiology, Meander Medical Center, Amersfoort, the Netherlands

**Keywords:** Lung cancer, Observer variation, Cancer screening, X-ray computed tomography, Solitary pulmonary nodule

## Abstract

**Objectives:**

Lung-RADS represents a categorical system published by the American College of Radiology to standardise management in lung cancer screening. The purpose of the study was to quantify how well readers agree in assigning Lung-RADS categories to screening CTs; secondary goals were to assess causes of disagreement and evaluate its impact on patient management.

**Methods:**

For the observer study, 80 baseline and 80 follow-up scans were randomly selected from the NLST trial covering all Lung-RADS categories in an equal distribution. Agreement of seven observers was analysed using Cohen’s kappa statistics. Discrepancies were correlated with patient management, test performance and diagnosis of malignancy within the scan year.

**Results:**

Pairwise interobserver agreement was substantial (mean kappa 0.67, 95% CI 0.58–0.77). Lung-RADS category disagreement was seen in approximately one-third (29%, 971) of 3360 reading pairs, resulting in different patient management in 8% (278/3360). Out of the 91 reading pairs that referred to scans with a tumour diagnosis within 1 year, discrepancies in only two would have resulted in a substantial management change.

**Conclusions:**

Assignment of lung cancer screening CT scans to Lung-RADS categories achieves substantial interobserver agreement. Impact of disagreement on categorisation of malignant nodules was low.

**Key Points:**

*• Lung-RADS categorisation of low-dose lung screening CTs achieved substantial interobserver agreement.*

*• Major cause for disagreement was assigning a different nodule as risk-dominant.*

*• Disagreement led to a different follow-up time in 8% of reading pairs.*

## Introduction

The National Lung Screening Trial (NLST) demonstrated a decrease in lung cancer-specific mortality of 20% [1]. Together with follow-up research, this led to a recommendation of CT lung screening for eligible subjects by several organisations, including the US Preventative Services Task Force [2]. Lung cancer screening programs are now being implemented in the USA. Interpretation of CT scans for lung cancer screening is a labour-intensive task for radiologists and the assessment of malignancy risk in pulmonary nodules remains challenging. Various categorical management protocols and scoring systems have been developed to aid radiologists in the selection of high-risk nodules demanding a more invasive management [3–7]. Protocols are based on a combination of nodule type, nodule size, nodule growth, and other additional parameters such as subject characteristics and nodule morphology.

In 2014, the American College of Radiology (ACR) published the Lung-RADS Assessment Categories to standardise the CT lung screening reporting and management recommendations and facilitate outcome monitoring [4]. Lung-RADS contains five categories to differentiate high-risk from low-risk nodules using nodule type, nodule size and growth as criteria. For nodule type, solid is differentiated from subsolid nodule composition with the latter having a relatively higher malignancy risk [8]. Nodule size is determined using manual diameter measurements and growth is defined as an increase of at least 1.5 mm in diameter. The primary criteria for the various Lung-RADS categories are described in Table 1. A negative screening result corresponds to category 1 (negative) or 2 (benign appearance), while a positive screening corresponds to category 3 (probably benign) or 4 (suspicious). The last category is further divided into category 4A and 4B based on the probability of malignancy (5–15% or greater than 15%). Category 4X is a special category for lesions that demonstrate additional features or imaging findings that increase the suspicion of malignancy [4]. It is well known that both visual assessment of nodule type and manual diameter measurements suffer from substantial observer variability [9–13]. It is therefore of importance to evaluate how well radiologists agree on such a categorical system that uses manual size measurements and visual nodule classification as two major input parameters.

**Table 1:** Lung-RADS assessment category criteria

Lung-RADS category	Criteria for baseline CT scans	Criteria for follow-up CT scans	Management
1	No nodules, or nodules with complete, central, popcorn or concentric rings of calcification, fat-containing nodules	No nodules, or nodules with complete, central, popcorn or concentric rings of calcification, fat-containing nodules	Annual LDCT screening
2	SN < 6 mm PSN < 6 mm in total diameter GGN < 20 mm	SN and PSN < 6 mm SN new < 4 mm GGN < 20 mm or unchanged or slowly growing Category 3–4 nodules unchanged at ≥ 3 months	Annual LDCT screening
3	SN ≥ 6 and <8 mm PSN ≥ 6 mm in total diameter with solid component < 6 mm GGN ≥ 20 mm	SN new ≥ 4 and < 6 mm PSN new < 6 mm GGN new ≥ 20 mm	6 month LDCT
4A	SN ≥ 8 and < 15 mm PSN ≥ 6 mm with solid component ≥ 6 and < 8 mm	SN growing < 8 mm or new ≥ 6 and < 8 mm PSN ≥ 6 mm with new or growing solid component < 4 mm	3 month LDCT; PET/CT
4B	SN ≥ 15 mm PSN with a solid component ≥8 mm	SN new or growing and ≥ 8 mm PSN 6 mm with new or growing solid component ≥ 4 mm	Chest CT with/without contrast, PET/CT and/or tissue sampling

*SN* solid nodule, *PSN* part-solid nodule, *GGN* pure ground-glass nodule

The purpose of this study was to quantify the interobserver variability for applying the Lung-RADS Assessment Categories to subjects having undergone low-dose screening computed tomography (CT). Secondary outcome parameters were the effects of interobserver variability on patient management and test performance.

## Materials and methods

### Data

All study cases were derived from the NLST [14]. The NLST was approved by the institutional review board of all participating centres and all participants provided informed consent. This study has been registered by the NLST study board under number NLST-187.

### Assessment of Lung-RADS categories and study group

The NLST included 26,309 subjects that underwent at least one low-dose chest CT scan [14]. We received all scans (screening rounds T0, T1 and T2) from a random sample of 4512 subjects. The NLST database provides information regarding nodule type, total nodule size and lobe location. Information regarding lung cancer diagnosis is available for all participants over a median follow-up period of 6.5 years. In total, 6121 nodule annotations were recorded in the database for these 4512 subjects.

Lung-RADS categorisation of all scans in this data set was performed to be used as selection criteria later in this study. Lung-RADS categories were assigned to all 6121 nodule annotations in the 4512 subjects using the pre-existing annotations from the NLST database with respect to nodule type (solid/part-solid/non-solid) and size (average of long and perpendicular diameter on axial section). This was done by a researcher (Ph.D. candidate with an M.Sc. degree in medicine) and a chest radiologist with more than 20 years of experience. Since category 4X is based on subjective morphological criteria other than nodule type, size and growth, this category was disregarded in our study. The nodule with the highest Lung-RADS category determined the Lung-RADS category for the CT scan. For Lung-RADS categorisation of the sub-solid nodules listed in the NLST database, a medical student specifically trained in segmentation and classification of pulmonary nodules in screening CT scans semi-automatically determined the size of the solid component because this information is not provided by NLST. Note that this pre-study Lung-RADS categorisation is only used to select cases but did not serve as standard of truth.

To ensure a balanced representation of all Lung-RADS categories, we formed an enriched study group. Using the Lung-RADS categories described above, we randomly selected 20 scans per category 1/2, 3, 4A and 4B, respectively, out of the pool of 4512 participants. This was done separately for T0 and T1 scans. Lung-RADS categories 1 and 2 were grouped together. Thus, our final data set for the observer study consisted of 80 T0 scans, and 80 T1 CT scans with the corresponding 80 T0 scans from a total of 160 unique subjects.

### Observers and reading methodology

Three radiologists and four fifth-year radiology residents from five different medical centres participated in this study as observers. They had experience with pulmonary nodules and reading chest CT scans ranging from 4 to 30 years. One of them had experience with reading screening CTs.

A dedicated workstation was used (CIRRUS Lung Screening, Radboud University Medical Center, Nijmegen, the Netherlands) which allowed for evaluating the complete CT scan in all three projections with interactive viewing tools such as magnification, manual diameter measurements and adjustment of window settings. Tools such as computer-aided detection (CAD) marks, volumetry or automatic linking of T0 and T1 scans were specifically disabled to mimic reading in a PACS environment without dedicated computerised applications. Nodule annotations made by readers were stored by the workstation in a local database. Readers were not informed about the selection or distribution of Lung-RADS categories within the study group.

For the *baseline* scans, observers were asked to assess the complete CT scan, to define the risk-dominant nodule, select the nodule type (solid, part-solid, pure ground-glass or calcified) and measure the longest and perpendicular diameters on axial sections, which were subsequently averaged and rounded to the nearest whole number [4]. Then they were asked to categorise the CT scan into either Lung-RADS category 1, 2, 3, 4A or 4B on the basis of the risk-dominant nodule. Readers were not asked to annotate all nodules; it was left to the readers’ discretion to annotate and measure only a single or—if it was felt necessary—several nodules in order to identify the risk-dominant nodule.

For the *follow-up* cases, the T0 and T1 CT scans were shown next to each other on two separate monitors allowing the two scans to be reviewed side-by-side. T0 scans of the follow-up cases had been pre-read by the researcher and an expert radiologist both not involved in the observer study. Their annotations and Lung-RADS categories were available to the observers while they were asked to categorise the follow-up scans.

All observers read all cases in different random order in at least two reading sessions with unlimited reading time available. A printout with Lung-RADS categories was available during the reading. Prior to the first reading session, each reader individually studied a set of 24 training cases including multiple cases per Lung-RADS category to get familiar with Lung-RADS categorisation. For each case, the pre-existing NLST annotations and the Lung-RADS category calculated from them were available to the reader for feedback.

### Analysis of reading data

Since the NLST did not assign CT scans to a Lung-RADS category, there was no reference standard. For every case, it was verified if observers had assigned the correct Lung-RADS category to their own annotations; if not, such Lung-RADS assignment errors were documented and subsequently corrected by the researcher on the basis of the observer’s own nodule annotations. Linearly weighted Cohen’s kappa statistics was utilised to determine pairwise interobserver agreement for the Lung-RADS categorisation of each CT scan. Pairwise kappa values were averaged over all possible observer pairs resulting in a mean kappa with a 95% confidence interval (CI). Kappa values were interpreted using the Landis and Koch guidelines [15]. Descriptive statistics were used where appropriate. Discrepant readings were subdivided into two groups dependent on whether the same or different nodules were assigned as being risk-dominant. Only same-nodule discrepancies were analysed and assessed for variation in the assignment of nodule type, assessment of growth or categorical difference in absolute diameter measurement.

To quantify the impact of reader variability on the actual test performance we assessed the observer variability for assigning baseline (T0) scans into screening-negative (Lung-RADS categories 1/2) or screening-positive scans (Lung-RADS categories 3, 4A or 4B) similar to Pinsky et al. [16] and McKee et al. [17].

Secondly, to assess the impact of observer disagreement on actual subject management, a distinction was made between minor and substantial management disagreement. A substantial management discrepancy referred to a difference in follow-up time of at least 9 months and occurred for disagreement between Lung-RADS categories 1/2 and 4A or 4B, respectively. Minor management discrepancies referred to a difference in follow-up of 6 months at maximum and occurred for disagreement between Lung-RADS categories 1/2 and 3, between categories 3 and 4A or 4B, respectively, or between categories 4A and 4B, respectively. Numbers and percentages are reported.

## Results

In 6% of all scores (68/1120), observers assigned the wrong Lung-RADS category to their own annotations. Those assignment errors were revised on the basis of the reader’s personal annotations of nodule type, size and growth. For the seven observers, it occurred on average in 8.5 cases with a range between 3 and 19.

### Interobserver agreement

Interobserver agreement for the Lung-RADS categories was substantial with a mean weighted kappa of 0.67 (95% CI 0.58–0.77) averaged over all observers. Weighted kappa values varied from 0.63 (95% CI 0.53–0.73) to 0.73 (95% CI 0.64–0.81) for the observer pairs, all being substantial.

Interobserver agreement was slightly higher for baseline scans with a mean pairwise kappa of 0.70 (95% CI 0.58–0.82), compared to 0.63 (95% CI 0.49–0.77) for the follow-up scans.

### Causes of Lung-RADS disagreement

When considering all possible reading pairs among the seven observers (21 pairs × 160 scans = 3360 observations), disagreement with respect to CT categorisation was observed in about one-third (971/3360, 29%) and resulting in substantial management difference in 8% of all reading pairs (278/3360).

Reading discrepancies were divided into those related to the same risk-dominant nodule and into those related to different risk-dominant nodules.

**Fig. 1:**
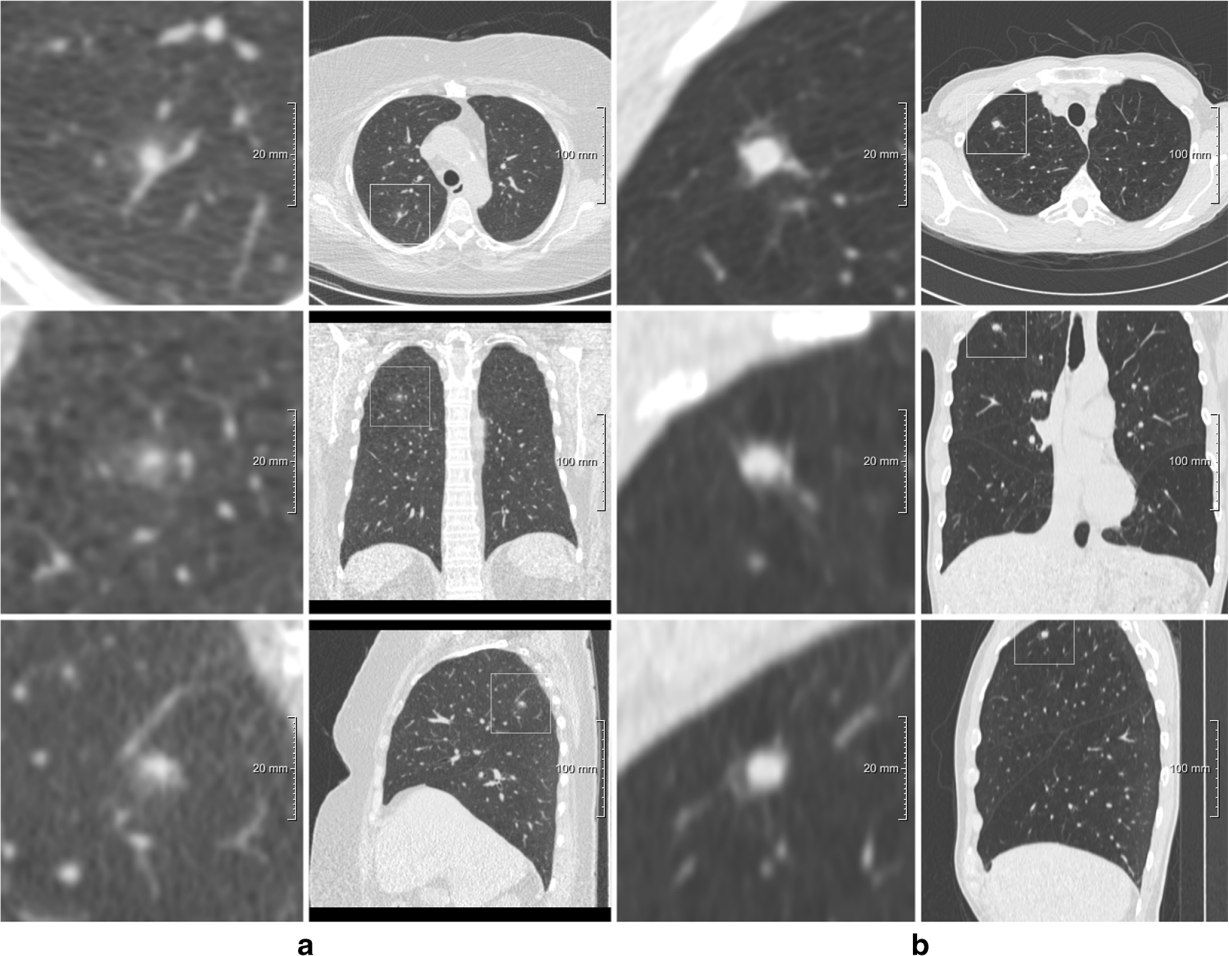
Two examples of risk-dominant nodules characterised differently by the seven observers which led to Lung-RADS classification differences. Each example shows a nodule displayed in magnified view (left column, field of view of 60 × 60 mm) and normal view (right column). The three different rows show axial (top), coronal (middle) and sagittal (bottom) plane. **a** T1 CT scan with a nodule that was classified as Lung-RADS 2 by one observer (new small solid nodule), Lung-RADS 4A by one observer (new part-solid with solid component < 4 mm) and Lung-RADS 4B by five observers (new part-solid, with solid component > 4 mm). **b** T1 CT scan with a nodule that was classified as Lung-RADS category 4A or 4B by five observers (new solid nodule with a measured diameter ranging from 7 to 9.6 mm) and Lung-RADS category 4B by two observers (new part-solid nodule with a solid component > 4.0 mm)

Discrepancies related to the same risk-dominant nodule turned out to be the minority with 26% (250/971, 47 cases), in which the two observers assigned different Lung-RADS categories on the basis of differences in nodule size measurements (207/971, 21%), nodule type classification (37/971, 4%) or growth assessment (6/971, 1%). This led to substantial discrepancies with respect to case management in only one case (Lung-RADS 1/2 versus 4A). This specific case is shown in Fig. 1.

The majority of pairwise disagreements (721/971, 74%), however, were caused by assigning different nodules as risk-dominant. Substantial management discrepancy between categories 1/2 and 4A or 4B occurred in 38% (277/721) of them and occurred in 48 of the 160 subjects. In the majority of those cases (77%, 553/721) the readers annotated only one nodule, namely the risk-dominant one of his/her choice. In the minority of cases (23%, 168/721) observers annotated two nodules but assigned a different risk stratification as a result of variations in nodule type classification (47/168, 28%), diameter measurement or growth assessment (121/168, 72%).

**Fig. 2:**
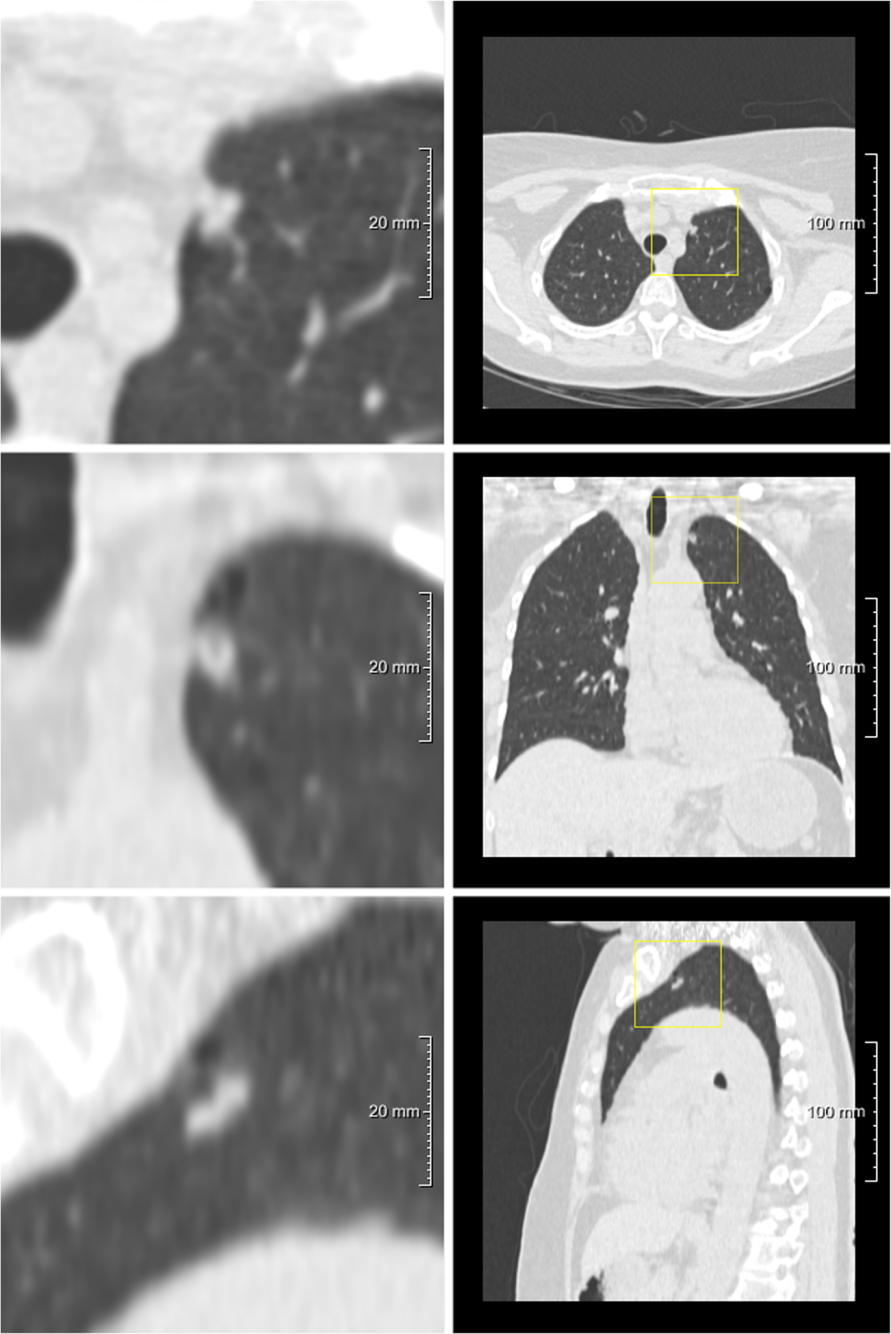
One example of a risk-dominant nodule characterised differently by the observers which led to Lung-RADS classification differences with impact on subject management within one observer pair. Each example shows a nodule displayed in magnified view (left column, field of view of 60 × 60 mm) and normal view (right column). The three different rows show axial (top), coronal (middle) and sagittal (bottom) plane. This was a benign nodule detected on a T0 scan and was classified as Lung-RADS 4A by one observer (solid nodule with a measured diameter of 9 mm), Lung-RADS 3 by five observers (solid nodule with measured diameters of 6 or 7 mm) and Lung-RADS 2 by one observer (solid nodule with a measured diameter of 5 mm)

Details are provided in Table 2. Figure 2 show examples of cases where observers disagreed for various reasons.

**Table 2:** Factors of disagreement in Lung-RADS category assessment on observer basis

Cause of observer disagreement	Number
Same risk-dominant nodule	250 (26%)
Interpretation: different nodule type	37 (15%)
Interpretation: nodule diameter measurement	207 (83%)
Interpretation: nodule growth	6 (2%)
Different risk-dominant nodule	721 (74%)
Total	971 (100%)

### Impact of nodule size on Lung-RADS disagreement

No correlation was seen between nodule size and reader disagreement. In 94 of the 160 study subjects at least one discrepant reading pair was seen. Only low-risk and thus smaller nodules (categories 1/2 or 3) were recorded in 29 subjects, and only higher-risk and thus larger nodules (categories 3, 4A and/or 4B) were recorded in 17 subjects. In the remaining 48 subjects a mix of low- and higher-risk nodules was recorded.

### Impact of observer variability on test performance

For the seven observers the mean percentage of screening-positive scans out of all scans was 53% (86/160) with a range between 44% and 61%. Correspondingly the screening-negative scan rate was 47% (74/160) with a range between 39% and 56%. Observer pairs differed on average in 12/160 cases (8%) with a range of 1–27 cases between those screening positive or negative.

According to the NLST database, in 13 cases lung cancer had been diagnosed in the same year as the CT scan included in this study. Pooled over all seven observers, the CT scans of these 13 subjects were classified as Lung-RADS category 4B in 78% (71/91) and as Lung-RADS 4A in 18% (16/91). The remaining four classifications referred to the same scan and included Lung-RADS 3 (*n* = 2) and Lung-RADS 1/2 (*n* = 2), all of them referring to a non-malignant nodule in the same scan while the actual malignant nodule was not perceived as the risk-dominant lesion.

## Discussion

The diagnostic and economic success of a lung cancer screening program will depend on accurate and reproducible differentiation between high-risk nodules, requiring more intense work-up, and low-risk nodules. Therefore, the ACR developed the Lung-RADS Assessment Categories to support radiologists in their decision-making by standardising lung cancer screening CT reporting and management recommendations [4]. The Lung-RADS categories in their current format are based on visual nodule type classification, manual nodule size and documentation of growth. For both reading tasks substantial interobserver variability has been reported previously [9–13]. The goal of our study was therefore to quantify interobserver variability for Lung-RADS categorisation of low-dose screening CTs and to assess its impact on test performance and subject management. To ensure adequate representation of all Lung-RADS categories we used an enriched study group that included baseline and follow-up CTs.

We found an overall substantial pairwise inter-reader agreement of Lung-RADS categorisation of screening CT scans, which underlines the value of this categorical system in harmonising interpretation and management of screening CTs. Agreement was slightly higher for baseline scans than for follow-up scans (kappa 0.71 versus 0.63). This finding might be explained by the fact that for follow-up the complexity of visual assessment including comparison and determination of nodule growth is higher than for baseline alone.

Variability of Lung-RADS categorisation may refer to the same risk-dominant nodule or to assignment of different nodules as risk-dominant. The first was less common (26%) and most importantly had only very rarely a substantial impact on subject management (0.4%). The latter was seen much more frequently (74%) and led to a management difference (≥ 9 months difference in follow-up time) in 8% of all reading pairs. Interestingly, not only measurement and classification differences were responsible for these discrepancies but apparently also differences in nodule perception given the fact that in the majority of different nodule categorisation the readers selectively annotated their risk-dominant nodule of choice. We did not ask the observers to annotate all nodules detected but left it to their discretion which nodules would be measured. While in the NLST trial, annotation of all nodules larger than 4 mm was requested, no recommendation is made in Lung-RADS concerning this issue. Therefore it remains open to what extent differences in detection or characterisation contributed to the reader variability. Similarly, Pinsky et al. reported earlier that nodule detection and documentation substantially varied between screening radiologists in the NLST trial [18]. Another factor that may have contributed to disagreement is the small separation between Lung-RADS categories. For example, the difference between a Lung-RADS 2 solid nodule and a Lung-RADS 4A solid nodule is only 2.1 mm (5.4 mm is Lung-RADS 2, 7.5 mm is Lung-RADS 4A). This “closeness” of the Lung-RADs categories may also explain why despite relatively frequent disagreements, only a small proportion had effects on patient management.

The primary objective of this observer study was to quantify variability of Lung-RADS categorisation without special focus on actual malignancies. Subjects were randomly included in the study group to ensure a balanced distribution of all Lung-RADS categories, and consequently the number of scans with malignancies diagnosed in the year of the scan was limited (*n* = 13). The actual histology of these malignancies is unknown to us. Nevertheless though the number of discrepancies with substantial management impact seems not negligible, it has to be underlined that the number of discrepancies was low in these 13 CTs. With the exceptions of four readings, the malignant nodules were categorised as Lung-RADS 4A or 4B, resulting in intensive further diagnostic work-up, and for only two readings the discrepancy resulted in a potential delay of more than 9 months.

All observers read 24 cases prior to reading the study data set in order to become familiar with the Lung-RADS definitions. They also had a printout of the original Lung-RADS assessment rules available during their reading. Nevertheless, wrong assignment of the Lung-RADS category criteria occurred in 6% of all readings and in 8.5 cases on average per observer. Since the main goal of our study was to investigate inter-reader variability as a result of different nodule interpretation, we adjusted incorrect Lung-RADS categories to the observer’s own nodule annotations before data analysis. However, wrong assignment of the Lung-RADS category may turn out to be a problem in practice as well. Computerised tools that automatically assign the correct Lung-RADS category of a scan once the pertinent data of one or more nodules have been entered may therefore prove useful [19].

Our study has some limitations. Reader experience plays an important role in observer studies. To capture a realistic estimation of the extent of observer variability and its impact on patient management, we included a broad range of observers with and without experience reading actual screening CTs. All readers, however, were well trained in thoracic CT and skilled in interpreting nodules, thus representing radiologists potentially involved in screening in the future. Parts of the observer variability, especially with respect to identification of the risk-dominant nodule, might still be related to lack of experience, suggesting that dedicated training is important, as also articulated by the ACR [20]. Interestingly, no significant differences in agreement were observed between residents and radiologists.

Other limitations are related to the study design. We used an enriched cohort consisting of 160 cases categorised as Lung-RADS category 1/2, 3, 4A and 4B on the basis of our algorithm. We chose this approach to be able to draw meaningful conclusions over the whole spectrum of nodules. This, however, means that our results need to be interpreted in the light of the enriched study group and cannot simply be extrapolated to an unselected screening cohort.

Secondly, the Lung-RADS category 4X was not considered in this study. This category gives radiologists the opportunity to upgrade a Lung-RADS category 3 or 4A nodule to category 4X on the basis of suspicious morphological findings and resulting in intensified possibly invasive diagnostic work-up. In addition to quantitative measures it adds subjective assessment of nodule morphology which we aimed to exclude from our analysis.

Thirdly, no reference standard was available for this data set, since Lung-RADS was not used in the original NLST annotations. As our study focuses on the effect of interobserver variability and its impact on management no reference standard was required.

Fourthly, since we did not ask our readers to annotate all identifiable nodules, we were not able to investigate whether the assignment of different nodules as risk-dominant was caused by an error in detection or an error in characterisation. In future studies, this should be taken into account.

Lastly, we defined a difference in follow-up of at least 9 months as a substantial impact on patient management. However, whether observer variations would have an impact on tumour stage and eventually patient outcome remains open.

In summary, the Lung-RADS Assessment Categories achieved substantial interobserver agreement. Disagreement was mainly caused by assigning a different risk-dominant nodule. In our enriched cohort disagreement led to different follow-up interval of more than 9 months in 8% of all reading pairs with little effect on the diagnosis of the malignancies within this series. The use of (semi-)automatic detection, segmentation and classification tools would likely reduce disagreement amongst readers, but the availability of these tools in clinical practice is still low and they require careful standardisation.

## Abbreviations and acronyms

ACR: American College of Radiology
CAD: Computer-aided detection
CI: Confidence interval
CT: Computed tomography
NCI: National Cancer Institute
NELSON: Dutch-Belgian Lung Screening Trial
NLST: National Lung Screening Trial
